# Potential Therapeutic Benefit of NAD^+^ Supplementation for Glaucoma and Age-Related Macular Degeneration

**DOI:** 10.3390/nu12092871

**Published:** 2020-09-19

**Authors:** Gloria Cimaglia, Marcela Votruba, James E. Morgan, Helder André, Pete A. Williams

**Affiliations:** 1 Department of Clinical Neuroscience, Division of Eye and Vision, St. Erik Eye Hospital, Karolinska Institutet, 112 82 Stockholm, Sweden; CimagliaG@cardiff.ac.uk; 2 School of Optometry and Vision Sciences, Cardiff University, Cardiff CF24 4HQ, Wales, UK; VotrubaM@cardiff.ac.uk (M.V.); morganje3@cardiff.ac.uk (J.E.M.); 3 Cardiff Eye Unit, University Hospital Wales, Cardiff CF14 4XW, Wales, UK; 4 School of Medicine, Cardiff University, Cardiff CF14 4YS, Wales, UK

**Keywords:** nicotinamide adenine dinucleotide, glaucoma, age-related macular degeneration, mitochondria, retina, optic nerve, retinal pigment epithelium

## Abstract

Glaucoma and age-related macular degeneration are leading causes of irreversible blindness worldwide with significant health and societal burdens. To date, no clinical cures are available and treatments target only the manageable symptoms and risk factors (but do not remediate the underlying pathology of the disease). Both diseases are neurodegenerative in their pathology of the retina and as such many of the events that trigger cell dysfunction, degeneration, and eventual loss are due to mitochondrial dysfunction, inflammation, and oxidative stress. Here, we critically review how a decreased bioavailability of nicotinamide adenine dinucleotide (NAD; a crucial metabolite in healthy and disease states) may underpin many of these aberrant mechanisms. We propose how exogenous sources of NAD may become a therapeutic standard for the treatment of these conditions.

## 1. Introduction

Glaucoma and age-related macular degeneration (AMD) account for the majority of irreversible blindness cases in the elderly [1,2]. These diseases share common pathophysiological processes, such as oxidative stress, inflammation, and mitochondrial dysfunction. Glaucoma is characterized by the progressive dysfunction and loss of retinal ganglion cells (RGCs; the output neuron of the retina), thinning of the retinal nerve fiber layer (RNFL; the axons of RGCs), cupping of the optic disc and optic nerve degeneration resulting in irreversible blindness [3]. Glaucoma can be divided into two main categories, open-angle and angle-closure, depending on the anatomy of the anterior chamber angle. Despite morphological variations and the pathological progression rate, both are associated with increased intraocular pressure (IOP or ocular hypertension), the only current clinical therapeutic target [4]. While elevated IOP is the principal therapeutic target, glaucomatous damage can arise in eyes with IOPs in the normal range (also referred to as normal tension glaucoma, NTG) [5], although there is evidence that IOP lowering strategies have therapeutic benefit in some of these cases [6].

In AMD, the causative pathology lies in the outer retinal layers characterized by photoreceptor and retinal pigment epithelium (RPE) degeneration resulting in central vision loss [7]. One of the hallmarks of AMD is drusen, collections of degeneration material lying at the level of the RPE/Bruch’s membrane interface [8]. These deposits under the macula are often used to identify the stage of the pathology. AMD is clinically classified based on the presence of exudate as either the dry (atrophic) form or the wet (neovascular) form [9]. Dry AMD, which is the most frequent, displays a slow progressive dysfunction of RPE, photoreceptor loss, and retinal degeneration, while wet AMD, which is more aggressive and has a faster progression, presents as choroidal neovascularization with either intraretinal or subretinal leakage, hemorrhage, and RPE detachment [10,11]. There has been intense interest in the development of more effective therapies to arrest the progression of AMD. The U.S. National Library of Medicine currently lists that 272 out of 1716 AMD clinical trials are still active worldwide [12]. There is some evidence that dietary supplementation or nutraceuticals can be beneficial in some types of AMD. The Age-Related Disease Study (AREDS) demonstrated that a daily supplementation of an antioxidant cocktail (e.g., vitamin C, vitamin E, zinc and cupric oxide, and β-carotene) to be effective in delaying the progression of intermediate to advanced AMD. In a second study, AREDS2, β-carotene was substituted with lutein due to the increase incidence of lung cancer related to that supplement administration in current or former smokers [13]. The most effective treatment, albeit exclusively used for wet AMD, has come with the use of monoclonal antibodies which are injected intravitreally to arrest the effect of vascular endothelial growth factor (VEGF) in the development of subretinal neovascularization. Although anti-VEGF is the current mainstay approved treatment, it is not able to reverse vision already lost and to impede recurrences of CNV [14].

Both glaucoma and AMD share many common underling mechanisms. In this context, nicotinamide adenine dinucleotide (NAD^+^) has received considerable interest as a neuroprotective agent. NAD^+^ is a key molecule in healthy neurons and plays central role in cellular bioenergetics [15]. Its age-related depletion affects the biological coping mechanisms associated with inflammation, oxidative stress, and accumulation of damaged molecules [16]. All these alterations are intrinsically linked to retinal degenerative pathologies, such as glaucoma and AMD, and with compelling evidence that NAD^+^ supplementation would be a promising intervention in these diseases [17], alone or possibly in combination with other nutraceutical agents such as saffron [18,19] and zeaxanthin [20,21], or pyruvate [22], which together provide neuroprotection by targeting multiple disease mechanisms. This review focuses on the shared pathophysiological mechanisms of glaucoma and AMD and the potential role that NAD^+^ may play in their mitigation.

## 2. Nicotinamide Adenine Dinucleotide

### 2.1. NAD Biosynthetic Pathways

NAD^+^ was first reported in 1906 as a product of yeast fermentation [23,24]. The potential importance of NAD^+^ was revealed in 1937 when Joseph Goldberger identified that deficiency of vitamin B, the common name of nicotinic acid and a precursor of NAD^+^, as the cause for pellagra [25]. Diets high in corn and deficient in animal protein sources were implicated in the disease since animal protein is a major source of the essential amino acid tryptophan which is the only source for the de novo biosynthesis of NAD^+^. NAD^+^ is the last enzymatic product of three important biosynthetic pathways in the mammalian metabolism, the kynurenine (de novo), Preiss–Handler (from nicotinic acid), and salvage (recycling NAD^+^ from nicotinamide) pathways. The common limiting step for NAD^+^ synthesis is the enzyme nicotinamide mononucleotide adenylyltransferase (NMNAT) expressed as three protein isoforms (NMNAT1, NMNAT2, and NMNAT3) with differing cellular localizations [26]. These three protein variants have differential effects on cellular bioenergetics depending on the paralogue that is involved in the catalysis of NAD^+^ [27]. NMNAT1 and NMNAT3 are ubiquitously expressed, but in mammalian systems NMNAT1 is predominantly nuclear while NMNAT3 is mitochondrial. NMNAT2 localizes to the Golgi and cytosolic compartments and localized exclusively to neurons and hair follicles [28]. In neurons, NMNAT2 plays a pivotal role in axon development and survival [29,30,31] and its depletion can trigger Wallerian degeneration [32,33]. While the role of NAD^+^ in balancing cellular homeostasis is evolutionally conserved [34], only three pathways that lead to NAD^+^ synthesis have been described (in mammalian cells, the core focus of this review) [35]. De novo NAD^+^ synthesis initiates with the amino acid tryptophan (TRP) which is enzymatically degraded into kynurenine, which after a series of reactions leads to NAD^+^ [36]. The Preiss-Handler and the salvage pathways are the other sources of NAD^+^ [37,38,39]. The Preiss-Handler pathway comprises three enzymatic steps that start with the phosphoribosylation of nicotinic acid and end with the amidation of nicotinic acid adenine dinucleotide into NAD^+^ [40]. The salvage pathway is responsible for 80% of cellular NAD^+^ production through the action of nicotinamide phosphoribosyltransferase (NAMPT) and NMNAT 1, which convert nicotinamide (NAM), or other NAD^+^ precursors such as nicotinamide riboside (NR), into NAD^+^ [39,41]. NAMPT first converts transforms NAM into nicotinamide mononucleotide (NMN) which is then aminated to NAD^+^ by NMNATs. The salvage pathway maintains the cellular NAD^+^ pools by recycling NAD^+^ from multiple sources. Once consumed, NAD^+^ is reinserted in the pathway as NAM for subsequent enzymatic modfication and recycling to NAD^+^ [42,43,44]. Besides NMNATs, three other class of molecules are worth mentioning: sirtuins (SIRTs), poly(ADP-ribose) polymerases (PARPs), and CD38. These enzymes require NAD^+^ as a substrate for their activities thus directly contributing to the delicate balance of NAD^+^ homeostasis [45]. SIRTs are involved in neuroprotection, neuronal development, survival, cell senescence, mitochondrial homeostasis, and cellular turnover. PARPs are crucial for DNA repair mechanisms and cellular stress responses [46]. CD38 is involved in aging, inflammation, and in some circumstances degrades NAD^+^ precursors [47].

### 2.2. NAD^+^ in Aging and Neurodegeneration

Biologically, physiological aging is a highly regulated process in which damage is taken into account but mitigated by compensating cellular processes. Conversely, uncontrolled aging, senescence, and degeneration irrevocably alter several cellular functions. Compelling evidence has identified a link between NAD^+^ and aging, and, although cellular NAD^+^ decline with age is normal, disruptions in NAD^+^ homeostasis are associated with aging pathologies as well as age-related diseases. As a proof of concept, many experiments conducted using model systems such as yeast, *C. elegans*, *D. melanogaster*, mice, and human cells have demonstrated that NAD^+^ replenishment can extend life span and health span [48,49,50,51]. Furthermore, the connection between central nervous system (CNS) impairment and NAD^+^ deficiency has been documented since the discovery of pellagra, in which neuro-dysfunction is one of the early manifestations of the disease [52]. This physiological link has been clarified further by evidence that NAD^+^ is one of the key molecules involved in neuroprotection, neuroinflammation, mitochondrial function, and adaptive responses to oxidative stress [15,26]. NAD^+^ pools during aging are deeply affected by PARPs, CD38 increased activity, and mitochondria SIRT3 loss of function [53]. Mitochondrial dysfunction due to lowered NAD^+^ bioavailability is also a common feature of Alzheimer’s disease (AD), Parkinson’s disease (PD), glaucoma, and AMD [54,55].

Mitochondria are not only the cell’s energy source but also regulate several cellular metabolic responses such as oxidative stress and calcium homeostasis, take part in the unfolded protein response (UPR^mt^) [56], and regulate apoptosis [57]. As such, their dysfunction or insufficiency dramatically affects cellular health and homeostasis [58]. All these functions are closely related to NAD^+^ availability. The efficient mitochondrial ATP production is mediated by oxidative phosphorylation system (OXPHOS), which is composed by multimeric complexes (Complexes I–IV), coenzyme Q, cytochrome C, and the F_0_-F_1_ ATPase (Complex V) [59]. Mitochondrial DNA (mtDNA) encodes for 13 structural subunits of Complexes I, III, IV, and V while the nuclear DNA (nDNA) for the further genes required for mtDNA maintenance, replication, transcription, translation, post-translational modification, transport, and assembly [60]. The probability of deficits in these pathways dramatically increases with age, and if damaged mitochondria are not cleared the remaining organelles cannot effectively compensate for the loss of capacity in the OXPHOS pathway [61]. This results in an excess ROS production due to the electron leakage along the respiratory chain [62]. The associated oxidative stress triggers secondary downstream deficiencies manifest as an NAD^+^ imbalance or alteration in Ca^2+^ homeostasis [63]. Mitochondrial Ca^2+^ accumulation enables the opening of the mitochondrial permeability transition pore (MPTP), which induces the release of pro-apoptotic molecules such cytochrome C and caspases that activate the intrinsic apoptotic pathway [64]. The adverse oxidative state that results from mitochondrial dysfunction can provoke a vicious cycle that can drive continued damage due to increased ROS production triggering an extended inflammatory reaction that intensify the damage. ROS acts as signaling molecules in several intracellular signaling pathways that help to maintain a physiological redox state when there is a balanced cellular crosstalk. When this communication is dysregulated by oxidative stress, ROS enhance the secretion of proinflammatory molecules [65]. The release of proinflammatory cytokines in the CNS activate the resident cells (microglia, astrocytes, and Müller cells) which secrete other proinflammatory cytokines (e.g., TNFs and INFγ) and interleukins that exacerbate the situation [66]. NAD^+^ can be directly or indirectly implicated in all of these mechanisms.

With age, a loss of genome integrity alters both mtDNA and nDNA. DNA damage is common and, if left unrepaired, can adversely impact cellular homeostasis. The ability to counteract this relies on SIRTs and in particular SIRT7, which plays a key role in the DNA damage response. This mechanism is strictly dependent on NAD^+^ availability and when NAD^+^ bioavailability is limited these enzymes cannot exert their function [67]. Mitochondria integrity is strictly dependent on this mechanism, and mtDNA mutations alter the efficiency of the electron transport chain, generating excess ROS and limiting mitochondrial biogenesis [68]. Experiments on Deletor mice, a mouse model of mitochondrial myopathy that carries a mtDNA replicative helicase mutation, have demonstrated that increasing NAD^+^ by daily administration of nicotinamide riboside (NR) was sufficient to restore mitochondrial function and to increase mitochondrial turnover [69,70]. Mitochondrial integrity is further protected by UPR^mt^, which either through an accurate mitochondrial biogenesis program or metabolic adaptation promotes cellular viability and mitochondrial recovery [71]. In an oxidative state, this system damages to the UPR^mt^ which increases with excessive ROS production due to Complex I and III impairment, mtDNA mutation [71], and excess levels of unfolded or damaged proteins that overcome the mitochondrial chaperone protein-folding capacity [72]. NAD^+^ regulation of SIRT3 is implicated in UPR^mt^ proper function via three diverse mechanisms that either promote mitophagy or activate antioxidant responses, and alternatively directly reduce protein misfolding and the accumulation of aggregates in the mitochondria [73,74]. Finally, NAD^+^ can act as a very powerful anti-inflammatory molecule. Evidence suggests that this effect is achieved through PARP1 activation and the inhibition of SIRT1. When the activity of PARP1 is inhibited due to reduced NAD^+^ availability, this event triggers a detrimental cellular cascade modulated by multiple pro-inflammatory responses. When NAD^+^ is replenished by NAM administration, PARP1 responds to the stimulus and can antagonize SIRT1 activity. This concert of mutual conditioning due to NAD^+^ results in the suppression of inflammatory mediators [75].

It is not surprising that interventions to boost NAD^+^ bioavailability are of great interest, especially considering the impact that this may have on neurodegenerative disease. Compelling evidence indicates that deficits in NAD^+^ production or processing may be drivers of ocular degenerations.

### 2.3. NAD^+^ and Eye Disease

The retina is one of the most energy demanding tissues requiring a constant energy supply and bioenergetic homeostasis to preserve function. In the last decade, the role of NAD^+^-related impairment has been highlighted in almost all retinal neurodegenerative disorders [76]. Direct acknowledgement of NAD^+^ retina related pathology comes from studies on Leber congenital amaurosis 9 (LCA9), a very severe retinal dystrophy that lead to complete vision loss caused by mutations in *NMNAT1* [77]. Around ten mutations in the *NMNAT1* gene have been identified in genome sequencing of LCA9 carriers [78,79] and extensive studies on LCA9 retinas have elucidated that NMNAT1 is not only responsible for NAD^+^ biosynthesis but is required for retinal structure, development, and function [80,81]. Photoreceptors damage due to NMNAT1 dysfunction is one possible deleterious mechanism demonstrated in AMD. In mice, a specific deletion of *Nampt* in rod and cones demonstrated the direct influence of NAD^+^ metabolism on photoreceptor survival and visual function, highlighting the strong connection with mitochondrial deficits in these cells [80]. However, photoreceptors are not the only cellular phenotype affected by NAD^+^ decline. In AMD, RPE cells relay on NAD^+^ energy supply to sustain their metabolic requirements. Rodent experiments with light-induced retinal damage and ex vivo retinas explant cultures of AMD mouse models have demonstrated that restoring normal NAD^+^ either prevents RPE-induced light insult or reduces the propagation of retinal damage [82,83,84]. Moreover, restoring NAD^+^ levels in AMD retinas decreased ROS RPE damage and significantly reduced the expression of proinflammatory molecules that are responsible for drusen formation [85,86]. Supporting this, experiments blocking NMNAT enzymatic activity promoted RPE detriment and induced a pathologic state that affected photoreceptors, outer and inner nuclear layer, and the plexiform layer [87,88]. In addition to AMD, glaucoma is profoundly connected to NAD^+^ functions. RGCs, the affected neuron in glaucoma, are energetically demanding and depend on NAD^+^. The view of glaucoma as a NAD^+^-related pathology has been derived from several scientific studies in which both NMNATs over-expression and NAD^+^-related dietary intervention have been involved. NMNAT1 is pivotal for axonal protection [89,90] and overexpression of NMNAT1 is a component of the Wallerian degeneration slow allele (*Wld^S^*) which delays Wallerian degeneration after axonal injury [91,92]. Furthermore, the NAD^+^ producing enzyme has been shown not only to protect RGCs axons but also somas in animal models of chronic and acute glaucomatous damage. In experiments using a mouse model with cytoplasmically located NMNAT1, cytNMNAT1-tg mice, NMNAT1 rescued RGCs from glaucomatous injury as well as from ischemic damage [93]. However, these neuroprotective effects are not only limited to NMNAT1. Overexpression of NMNAT3 rescued RGCs from ocular hypertension induced damage and prevented mitochondrial related insults [28,94]. Williams et al. in an study using DBA/2J mice, an extensively used mouse model for glaucoma, demonstrated that exogenous NAD^+^ in the form of nicotinamide dietary supplementation was capable to restore RGCs function in both aged and glaucoma-prone mice [95]. This work also demonstrated that viral gene therapy overexpressing *Nmnat1* in RGCs provided a significant neuroprotection, and when used in combination with nicotinamide administration rescued 94% of eyes from glaucomatous neurodegeneration [96]. This specific intervention protected RGCs soma, synapses, and axons, and prevented the loss of axoplasmic transport, demonstrating that many glaucoma deficits may be due to mitochondrial impairments derived by NAD^+^ disrupted metabolism [97]. RGCs axons are not the only neuronal compartment affected by the glaucomatous insults with RGC dendrites abruptly affected as well. Experiments conducted on *Drosophila* mutants overexpressing *Nmnat* have demonstrated that NMNAT is required to preserve dendritic structures, confirming the broad spectrum of NAD^+^ in the CNS [98].

## 3. Glaucoma

Glaucoma is characterized by the progressive degeneration of RGCs and their axons which make up the optic nerve resulting in visual field loss and irreversible blindness [99]. It is the major cause of irreversible blindness worldwide and its burden has been estimated to affect ~2% of the global population by 2040 [1]. To date, the only treatable and modifiable risk factor is IOP, however ~40% of treated glaucoma patients will still continue to be blind in at least one eye [4].

Clinically, glaucoma is divided into primary (open angle and angle closure glaucoma) and secondary (originating from trauma, medication, inflammation, cancer, or other primary conditions) glaucomas. Regardless of the initiating events, irreversible RGC neurodegeneration is the neuropathological hallmark of the disease. This specific class of neurons is located in the inner retina, with their axons making up the RNFL and the optic nerve. At the optic disc RGCs axons bundle together to exit the eye becoming the optic nerve, and at the level of the optic chiasm the optic nerve partially decussates to project to terminal visual thalami in the brain [100,101]. Changes in the RNFL and disc/cup ratio can be easily identified during an ophthalmoscopic examination [102]. However, it has been documented that RGCs death is preceded by several structural and functional changes in the dendrites, soma, and axons that hide from gross clinically detection [103]. This progressive and compartmentalized degeneration may offer a window for therapeutic interventions that can be useful to halt the underlying mechanisms of RGC neurodegeneration [104]. Structural changes of the dendrites, predominantly manifested with the pruning of the exterior branches, with a consequent impact on the neurotransmission due to synapses loss, can be detected early upon insult [105,106,107]. Dendritic architectural remodeling after IOP increase seems to be correlated not only with altered neuronal trafficking [108,109] but especially with complement activation [110] and mitochondrial impairments [111,112]. The spread of the axon damage is not linear and appears to initiate distally from the main site of injury [113], which has been proposed to be the optic nerve head (ONH) [114,115]. The mechanism by which the damage propagates along the axon is compartment-related [116] with several lines of evidence reported by Libby et al. in their studies of axonal degeneration [117,118]. These dramatic architectural changes that affect glaucomatous RGCs already illustrate the multiple components that are in charge of RGCs degenerative processes. As such, oxidative stress, inflammation, glial activation, neurotrophin deprivation, and excitotoxicity (among others) work in concert to drive RGC degeneration [119,120,121,122,123], with mitochondrial dysfunction being a potential driving force behind these multifactorial processes [124].

Efficient mitochondria are essential for RGCs survival, as evidenced in Leber’s hereditary optic neuropathy and autosomal dominant optic atrophy (which results in RGC degeneration and irreversible blindness) [61]. Mitochondria distribution along RGCs is asymmetric due to the different energetic demand among the RGC compartments, with a higher density in the dendrites and in the unmyelinated ONH [125,126], which are initial sites of neurodegeneration.

One of the first insults promoted by the mitochondria is the generation of a wide oxidative state in the cell and when ROS production overcomes the cellular tolerance or in response to other lethal stimuli, cytochrome *c* is released in the cytosol where in concert with other cytosolic factors forms the apoptosome and further promotes other caspase activation [127]. This process, the intrinsic apoptotic pathway, also activates astrocytes and microglia. In a normal state microglia and astrocytes secrete factors which are fundamental for RGCs health [128]. One of their roles as CNS resident cells is to sense metabolic alterations and to act in order to re-establish metabolic homeostasis, but in the presence of caspase 8 and 3 they become hyperactivated and start to release TNFα and several pro-inflammatory cytokines [4]. TNFα is the promoter of the extrinsic apoptotic pathway and is one of the main promoters of RGCs death. RGCs express TNF-R1, which is TNFα receptor, thus the bindings of the cytokine consequently induces RGCs apoptosis [129]. In addition, when Müller glia, microglia, and astrocytes are hyperactivated, their harm to RGCs is not limited to the apoptotic cascade but is also due to the cessation of their neuroprotection and neurotrophic support to the cells [130,131,132,133]. RGC deprivation of nerve growth factor (NGF) [134], brain derived neurotrophic factor (BDNF) [135], ciliary neurotrophic factor (CNTF) [136], and glial-cell line derived neurotrophic factor (GDNF) [137] have been well established as a connection with the glaucomatous damage. Cessation of the release of neurotrophic factors increases the intrinsic apoptotic cascade through the activation of mitogen-activated protein kinase (MAPK) pathway [138] inducing further mitochondrial dysfunction via BAX [139].

All these catastrophic events are likely to be linked to a decrease in NAD^+^ [140], and the lack of effective therapeutic strategies and the remarkable evidence of NAD^+^ involvement suggest a therapeutic role for NAD^+^ in glaucoma.

### Available Therapies

All the current available therapies are centered on IOP lowering. The first medical therapy to achieve IOP lowering was beta adrenoceptor agonists, which reduce the aqueous humor secretion blocking ciliary epithelium β-receptor [141]. These medications are normally once or twice daily but unfortunately are not free from adverse effects (e.g., interact with the heart rhythm or causing bronchospasm [142]) that sometimes might render the adherence to therapy difficult or even not recommended. To overcome this obstacle, rho kinases inhibitors therapies have been introduced, with some side effects reduction and quite few improvements in RGCs survival [143]. Unluckily, these therapies are not always effective and when they fail the only two alternative approaches are much more invasive and rely either on laser therapy or surgical interventions [144]. Both argon laser trabeculoplasty (ALT) and selective laser trabeculoplasty (SLT) or micropulse laser trabeculoplasty (MLT) aim to open the space at the trabecular meshwork [145]. Although sometimes effective at an IOP level, they may provoke an inflammatory state that, as discussed in the previous section, only aggravates the general pathological state. However, even this strategy can fail, resulting in surgery as a final option for many patients (e.g., trabeculectomy) [146].

## 4. Age-Related Macular Degeneration

Age-related macular degeneration is one of the leading causes of visual function impairments. One of the main risk factors, as implicated by the disease name, is aging, yet it is a multifactorial disease in which inflammation, metabolic dysfunctions, environmental factors, and genetic predisposition play a fundamental role. Clinically, AMD is classified as dry and wet, with the dry form accounting for 90% of cases and has a slower progression. The late and severe form of dry AMD is referred to as geographic atrophy (GA) and is characterized by a marked degeneration of RPE cells, photoreceptor loss, and degraded choriocapillaris [147]. Wet AMD accounts for the other 10% of cases and has a much faster progression and severe prognosis. At the latest stage, wet AMD shows abnormal vessels growth below the retina and that is why it is referred to as the choroidal neovascularization (CNV) form, with the CNV that breaks through the neural retina with an exudate of lipids, fluid, and blood [148]. One of the first signs that may suggest the progression to AMD is the development of drusen, yellow extracellular deposits that accumulated between the basal lamina of the RPE and the inner collagenous layer of the Bruch’s membrane. Their size and “softness” is one of the used parameters for identify the risk for developing AMD [149]; generally, small and hard drusen below 63 μm are considered as low risk, while drusen up to 125 μm are considered already an early sign of AMD. Hard drusen can become soft and more extended (above 125 μm), causing RPE abnormalities and spreading to the macula [150]. Until the 1970s, drusen were thought to be local deposits of lipids and carbohydrates but more recent knowledge has demonstrated the inclusion of elements of intra- and extraocular sources such as complement components and protein waste material, aligning with the involvement of several active processes participating in AMD development [151]. However, if we approach AMD with a molecular perspective, the pathological steps that drive photoreceptor apoptosis become clearer and provide a better understanding of this complex disease.

Oxidative stress is one of the leading pathological causes. For its structure and function, the retina is one of the highest oxygen-consuming organs, with the highest concentration of oxygen in the choroid that progressively decrease at the lower concentration in the outer segment of the retina [152]. This anatomical distribution generates an oxygen gradient along the retinal tissue that predispose to the photoreceptor oxidative damage [153]. Mitochondria are the first contributing factor to AMD oxidative stress, as elucidated from AMD animal models, some of which involve the knockdown of SOD2 (an isoform of the superoxide dismutase expressed in the mitochondria). Analysis of histological sections from these models showed a marked degeneration of the RPE with a conspicuous photoreceptor atrophy [154]. Other evidence of mitochondrial involvement came from Jones et al., who found specific mtDNA mutations in tissue samples of AMD patients [155,156]. Another study supporting a primary mitochondrial involvement in AMD comes from a study on primary culture of RPE cells from donors with AMD, wherein Ebeling et al. could demonstrate an improvement in mitochondrial function with a decrease in RPE cells loss upon Rapamycin and NAM treatment [157]. Mitochondrial dysfunctions significantly contribute to AMD related inflammation activating the nucleotide-binding oligomerization domain (NOD)-like receptor family or inducing the inflammasome activation, for example [158]. In response to the oxidative damage, macrophage infiltrates have been found in the subretinal space, in the choroid, and close to the drusen [159,160]. There are two subclasses of macrophages, M1 and M2; M2 are the anti-inflammatory macrophages that have been found to be predominant in the first pathological stage, performing a house-keeping role to minimize the damage, while M1, the pro-inflammatory macrophages, are the most conspicuous in the advanced stage and their role is to sustain and aggravate the inflammatory reaction, stimulating TNFα release, disrupting the balance among IL-1β and IL-18 that promote inflammasome related tissue-injury [161], and impairing autophagy and mitophagy [162]. Besides macrophages, complement activation also plays a substantial part in the AMD chronic inflammatory state. RPE cells constitutively express many complement pathway components that can be upregulated by both macrophages and microglia secreted molecules [163,164]. There are three main pathways that are involved in complement activation; all lead to C3 activation and the subsequent complement cascade. Complement factor H (CFH), which is an important regulatory protein that controls the activation of C3, has been found mutated in genetic screening of familial AMD, confirming the role of complement involvement in the pathogenesis of AMD [165].

Considering that AMD is a multifactorial disease and exists in two forms, the reduced oxygen supply from the choroid to the outer retina, namely retinal hypoxia, participates in the development of wet AMD [166]. Anatomical changes in the retina caused by accumulated drusen, increased Bruch’s membrane thickness or even the changing in the RPE layer, create a space between the choriocapillaris and the retina, thus forming a physical barrier which prevents an adequate oxygen supply. This mechanism stimulates VEGF to promote angiogenesis, but instead of restoring balanced oxygen levels it promotes edema with further retinal detachment that worsen the situation increasing hypoxia and VEGF production [167,168]. One of the factors responsible for VEGF production is the hypoxia-inducible factor-1α (HIF-1α). HIF-1α activity in normoxic conditions is inhibited but when the retina either detects an oxygen reduction or is stimulated by inflammatory mediators, such as TNFα, promotes VEGF expression [169,170]. The first study that was able to demonstrate the link between HIF-1α and VEGF expression in an animal model of ocular neovascularization was conducted by Lin et al. in 2012 [171]. In 2015, a pivotal study conducted by André et al. confirmed the same altered mechanisms in wet AMD. André et al.’s experiment demonstrated that following the induction of lesions mimicking CNV, an hypoxic state was detectable a few days after the insult, with the concurrent accumulation of HIF-1α in the tissue and subsequent upregulation of VEGF in the model’s neoangiogenic phase [172]. Furthermore, their experiment confirmed the hypothesis that HIF-1α expression was not correlated with the choroidal tissues but with the RPE cells [173,174].

The mechanisms that lead to vision loss in AMD act in concert and initiate a complex cycle that still needs to be completely clarified. Hanus et al. commented that it is still debatable if RPE cells loss is due to apoptosis or necrosis, which has broad implications on how we view and design treatments for AMD [175]. However, it was suggested by Rozing et al. that a two-level AMD pathological mechanism may offer a hypothetic therapeutic window prior to the development of the advanced disease stage. To simplify, they proposed that AMD might be firstly promoted by inflammatory and external modifiable factors (e.g., smoking, unhealthy lifestyle, and nutritional imbalance) that render the body susceptible to damage. Once weakened by these factors, the body cannot cope with the accumulated damage, and therefore reacts initiating the myriad of inflammatory responses aforementioned, firstly with the aim to repair the damage but then precariously progressing into a sustained chronic inflammation [176]. To that extent, although this extensive metabolic imbalance appears to be primarily focus on RPE cell loss, the cause of irreversible blindness is the secondary profound loss of photoreceptors, rod and cones, that cannot sustain this pronounced energy shortfall due to mitochondrial dysfunction and macrophage infiltration [177,178] (for an exhaustive review on these mechanisms, refer to [178]). In the absence of effective therapy, we hypothesize that we should develop preventive treatment for the first phase and curative treatment for the second phase.

### 4.1. Available Therapies

#### 4.1.1. Wet AMD

The treatment and management of AMD is as complex as the pathology, and thus far no effective disease modifying therapies for dry AMD have been validated. The first line approach for wet AMD targets the final common pathway in the development of CNV, namely VEGF. Anti-VEGF treatment seems to be effective in the majority of cases. The first treatment approved in 2004 by the FDA was pegaptanib, an RNA aptamer that binds the VEGF-165 isoform; ranibizumab, a newer Fab monoclonal antibody that inhibits the biologically active VEGF-A isoform has given better results on advanced CNV patients as assessed by the ANCHOR and MARINA trials [179,180]. Such antibodies were borrowed from the field of cancer treatment, as it was found effective in reducing angiogenesis in several carcinomas. Although effective the adverse side effects have led to the introduction of a new anti-VEGF treatment is 2011, aflibercept, that showed affinity to all forms of VEGF [7]. There are generally four therapeutic regimes adopted in clinical practice: fixed monthly or bimonthly regime of anti-VEGF intravitreal injections; a rata strategy; a treat-and-extend regimen; and an observe-and-plan regimen [181]. When these approaches fail, anti-VEGF treatments are used in combination with transpupillary thermotherapy, which delivers infrared spectrum wave lengths to target the tissue. In some cases, treatment with anti-VEGF is used in combination with steroids and photodynamic therapy [182,183]. Lastly, one of the newest approach for CNV AMD is pegpleranib, an inhibitor of platelet-derived growth factor (anti-PDGF), which promotes pericyte density decrease in the neovascular membrane to promote a beneficial environment for anti-VEGF agents [184]. Surgical procedures have been attempted with limited success, causing more harm than benefit. Both submacular surgery and macular translocation generally produce extensive hemorrhage and are associated with serious risks as retinal detachment or proliferative vitreoretinopathy [185,186].

#### 4.1.2. Dry AMD

The treatment available for dry AMD are very few and are mostly based on prevention and intervention at the earliest detectable pathological signs [148]. Fundamentally, the standard treatments relay on AREDS and AREDS2 recommendations because oxidative stress and inflammation are the triggering causes for GA AMD. Indeed, in a not yet approved therapeutic regime, patients with GA are receiving intravitreal injections of lampalizumab, a monoclonal antibody which selectively inhibit complement factor D. To date, in this MAHALO study, patients displayed a 20% reduction of the GA area, and, with continued intravitreal administration of the drug, in the CHROMA and the SPECTRI studies, the proportion of the area affected was reduced to 44% [187,188]. Since inflammation is considered the pivotal cause for the detrimental complement cascade, oral administration of tetracycline has provided some promising results [189,190]. In addition to those, there are ongoing clinical trials with possible lipid-lowering anti-inflammatory drugs (e.g., statins) and mitochondrial protective compounds such as MTP-131, commercially named Ocuvia, but unfortunately are still inconclusive [7]. Transplantation of photoreceptor and RPE cells derived from stem cells seem promising yet considerably invasive. Few patients have attempted this therapeutic approach, and the positive outcome suggested that, while less invasive treatments are not discovered, cell-based therapies will become the first line treatment for advanced dry AMD [191]. Other promising therapeutic concepts are based on targeting the inflammasome, downregulating cytokines, or finding an effective general neuroprotector but thus far nothing is available for an effective cure.

## 5. Supplementing NAD^+^ as a Therapeutic Approach/Strategy in Glaucoma and AMD

Supporting retinal health is at the core of maintaining retinal function. Despite the diversity of insults that can affect the retina, there are several common pathogenic mechanisms that may permit a unified therapy: mitochondrial dysfunction, oxidative stress, inflammation, and the inability to clear waste material from metabolic reactions. These are all mechanistically linked by the bioavailability of NAD^+^, either to insufficient production or over-consumption by NAD-consuming enzymes (e.g., SIRTs and PARPs) [192]. Given this, we propose increasing NAD^+^ as a putative effective therapeutic strategy for both glaucoma and AMD.

### 5.1. Potentials of NAD^+^ Intervention in Glaucoma

NAD^+^ increase, either by NAD^+^ precursor administration or gene therapy, is able to restore a normal phenotype and prevent age and disease related changes in the glaucoma mouse model [95,96,97]. The fact that NAD^+^ intervention does not target IOP but acts at the triggering pathological events allows nicotinamide (or other strategies to increase NAD^+^ pools) to be powerful tools in glaucoma treatment [193].

The decrease of NAD^+^ with age is physiological and may not always be related to disease processes directly. However, this appears not to be in the case in glaucoma. Supporting a hypothesis in which decreased levels of NAD^+^ render RGCs susceptible to glaucomatous neurodegeneration, Kouassi Nzoughet et al. demonstrated decreased sera NAD^+^ in a cohort of 34 POAG individuals [194]. A paper from Hui et al. [195] specifically highlights this point, translating animal and pre-clinical findings into clinical practice. In a clinical study of 57 glaucoma participants using inner retina visual function as the primary outcome measure, Hui and colleagues demonstrated a significant improvement in the visual function of existing glaucoma patients receiving 3 g/day nicotinamide for 12 weeks in addition to IOP lowering medication. Although the focus of glaucoma is mainly oxidative stress and inflammation, autophagic dysfunction, even if minor, plays a role in the pathology [124]. Accumulation of waste material in the lamina cribrosa (LC) and accumulation of lipofuscin have been found in LC donors cells with a consequence on dysregulated autophagic clearance at the level of LC with a clear impact on the ONH [196]. Targeting this other mechanism might be a problem considering the multitude of glaucoma correlated events if would not be treatable in the same way as the others, however NAD^+^ is strongly linked to autophagy suggests its utility here as well [197,198]. The neuroprotection afforded by nicotinamide may not only be due to improved mitochondrial function but also due to the NAD^+^/SIRT1 axis [199,200] or to protection from various other insults that affect RGCs, such as ischemia/reperfusion or light [201]. As such, further studies are needed to fully elucidate the role of nicotinamide and elevated NAD^+^ in healthy, diseased, and treated states.

### 5.2. Potentials of NAD^+^ Intervention in Age-Related Macular Degeneration

Restoring NAD^+^ might counteract some of the putative deficits that are most significant in the mechanisms for AMD development. Mitochondrial dysfunction appears to be the at bottom of the pyramid of AMD degenerative processes, not only due to mitochondrial involvement in oxidative stress but also as a dysfunctional organelle that has lost the capability to be replaced [158]. Mitochondrial homeostasis is regulated by mitochondrial biogenesis, fusion and fission, mitochondrial quality control, and mitochondrial autophagy (mitophagy). The process of mitophagy, in which damaged mitochondria are degraded and recycled, is pivotal for neuronal functions and the prevention of pathological neuronal dysfunction [202]. Impairments in this process have been associated with AMD and NAD^+^ may be a potential solution for the impaired mitophagy [203,204]. *PINK1* and *PARKIN*, which are responsible of the proper degradation of damaged mitochondria via mitophagy, appear to be upregulated in a mouse model of dry AMD, the erythroid 2-related factor 2/peroxisome proliferator-activated receptor gamma coactivator 1-alpha double knockdown, NFE2L2/PGC-1α^−/−^ mouse model [205]. The analysis of NFE2L2/PGC-1α^−/−^ RPE cells displayed an extensive upregulation of PINK and PARKIN levels along with an impaired mitochondrial clearance system, showing all the phenotypical change related to AMD [206]. PGC-1α governs the mitochondrial quality control system, and this function is enabled by the NAD^+^/SIRT1 axis. Restoring NAD^+^ levels compensate for the unbalanced SIRT1 activity re-establishing mitochondrial biogenesis, mitophagy, and UPR^mt^ [207]. Activating SIRT1 activity through NAD^+^ allows the deacetylation of several mitophagy-involved proteins such as Atg5, Atg7, and Atg8, bringing back or restoring to a normal level the mitochondrial quality control system [207,208]. Other lines of inquiry have reported that, instead of the NAD^+^/SIRT1/Atg5-7-8 axis, mitophagy is restored through the effect of NAD^+^/SIRT1/ULK1, confirming a profound involvement of this molecule in mitochondrial function [209]. Furthermore, the demonstration of the ability of NAD^+^ to halt the detrimental process in AMD comes from Saini et al. [210]. In this study, using human induced-pluripotent stem cells (hiPSC)-derived RPE cell line from AMD donors, NAD^+^ was capable of improving all the disease related features of AMD, such as drusen formation, complement-driven inflammation, mitochondrial dysfunction, and VEGF expression [210], supporting a hypothesis for NAD^+^ as a valuable treatment for both dry and wet AMD. Lastly, NAD^+^ has been shown to ameliorate another altered mechanism that affects RPE cells during AMD, a process called epithelial–mesenchymal transition (EMT). ETM consists in the loss of epithelial cell polarity and cell-cell adhesion and the acquisition of migratory and invasive properties as mesenchymal stem cells. This process RPE-ETM has been found to be linked to TGF-β upregulation, UPR^mt^ dysfunctions, yet, as reported by Zhou et al. and Hyttinen et al., NAM treatment prevented ETM in numerous RPE cell models, orchestrating the mechanism responsible for that shift [211,212].

### 5.3. Safety of NAD^+^ Supplementation

An increase in cellular NAD^+^ pools can be obtained from several exogenous sources including NR, NA, NMN, and NAM. Increasing evidence of the neuroprotective and positive aging effects of NAD^+^ precursors has intensified research concerning their safety level and side effects. All these precursors collectively fall under the umbrella of vitamin B_3_ replacement, even though their recommended intake is widely different. Although within therapeutic range, doses above 50 mg/day of NA are not advised due to flushing. Chronic intake of 3 g/day of NAM is well tolerated with side effects (mostly gastric minor disturbance) only in a small percentage of patients [213]. In classic studies from the 1950s and onwards, long-term higher doses of NAM at >9 g/day have been reported with minimal side effects, and some of the flushing side effects noted in these studies are likely from impure preparation, which include an admixture of NAM and NA [214]. NMN and NR are well tolerated as well, but the effects of long-term chronic administration are yet to be assessed fully in humans. One gram per day of NR was enough to improve both cardiovascular functions and lipid metabolism associated with increased NAD^+^ pool, which suggests tolerance, and possibly safety, at least at this dose level [215,216].

## 6. Conclusions

Glaucoma and AMD are common, irreversible, degenerative diseases with an urgent therapeutic need. The core of the deficits in these diseases may be reduced directly or indirectly to two main sources: physiological age-related NAD pool depletion and mitochondrial dysfunction. Supporting mitochondrial function is likely to be a promising strategy to inhibit neurodegenerative disease progression along with NAD^+^ repletion as an effective approach to restore normal function and cellular homeostasis. Recent evidence in human and animal models, as well as recent clinical trial evidence for nicotinamide treatment in glaucoma, highlights the potential for NAD^+^-related therapies for glaucoma and AMD, and potentially other age- and metabolism-related neurodegenerative diseases. The wide availability, affordability, safety, and tolerability of nicotinamide and other NAD^+^ precursors support further long-term clinical testing as well as potentially immediate clinical use.
